# Influence of tissue fixation on depth-resolved birefringence of oral cavity tissue samples

**DOI:** 10.1117/1.JBO.25.9.096003

**Published:** 2020-09-10

**Authors:** Karol Karnowski, Qingyun Li, Anima Poudyal, Martin Villiger, Camile S. Farah, David D. Sampson

**Affiliations:** a The University of Western Australia, Optical+Biomedical Engineering Laboratory, Department of Electrical, Electronic, and Computer Engineering, Perth, Western Australia, Australia; b Polish Academy of Sciences, Institute of Physical Chemistry, Warsaw, Poland; c The University of Western Australia, UWA Dental School, Perth, Western Australia, Australia; d Harvard Medical School, Massachusetts General Hospital, Wellman Center for Photomedicine, Boston, Massachusetts, United Sates; e Australian Centre for Oral Oncology Research and Education, Perth, Western Australia, Australia; f Fiona Stanley Hospital, Oral, Maxillofacial, and Dental Surgery, Murdoch, Western Australia, Australia; g University of Surrey, Surrey Biophotonics, School of Physics, Guilford, United Kingdom; h University of Surrey, Surrey Biophotonics, School of Biosciences and Medicine, Guilford, United Kingdom

**Keywords:** biomedical imaging, tissue fixation, optical coherence tomography, oral tissue

## Abstract

**Significance:** To advance our understanding of the contrast observed when imaging with polarization-sensitive optical coherence tomography (PS-OCT) and its correlation with oral cancerous pathologies, a detailed comparison with histology provided via *ex vivo* fixed tissue is required. The effects of tissue fixation, however, on such polarization-based contrast have not yet been investigated.

**Aim:** A study was performed to assess the impact of tissue fixation on depth-resolved (i.e., local) birefringence measured with PS-OCT.

**Approach:** A PS-OCT system based on depth-encoded polarization multiplexing and polarization-diverse detection was used to measure the Jones matrix of a sample. A wide variety of *ex vivo* samples were measured freshly after excision and 24 h after fixation, consistent with standard pathology. Some samples were also measured 48 h after fixation.

**Results:** The tissue fixation does not diminish the birefringence contrast. Statistically significant changes were observed in 11 out of 12 samples; these changes represented an increase in contrast, overall, by 11% on average.

**Conclusions:** We conclude that the fixed samples are suitable for studies seeking a deeper understanding of birefringence contrast in oral tissue pathology. The enhancement of contrast removes the need to image immediately postexcision and will facilitate future investigations with PS-OCT and other advanced polarization-sensitive microscopy methods, such as mapping of the local optic axis with PS-OCT and PS-optical coherence microscopy.

## 1 Introduction

Even though the potential of using optical coherence tomography (OCT) in the oral cavity and the oropharynx has been reported in a positive light,^1^^–^^3^ the evidence suggests that differentiation between healthy and potentially malignant oral tissue is still challenging when only conventional OCT image contrast is available. Some studies have been limited to only a few samples^2^ or have identified disease from a single feature extracted from OCT scans (e.g., epithelial thickness).^1^ In most studies reported thus far, diagnostic scores have been based on image comparison (OCT versus histology) by a previously trained surgeon and/or pathologist.^3^^–^^9^ The sensitivity and specificity of differentiating pathological from normal tissue reported in those studies are in the ranges 81.5% to 93.1% and 78% to 97.3%, respectively. The detection of carcinoma *in situ*, squamous cell carcinoma (SCC) versus noncancer (sensitivity: 93.1% and specificity: 93.1%), or SCC versus other pathologies (sensitivity: 93.1% and specificity: 97.3%) have also been reported.^7^ The study by Kraft et al.^10^ suggests the exact grading of oral dysplasia might be better determined if microlaryngoscopy is supported by examination by OCT. It is not clear, however, what the diagnostic accuracy would be if only OCT images were analyzed. The only study assessing the automatic classification of oral malignancy from OCT images alone was performed in an animal model (hamster cheek pouch).^11^ The performance of the sophisticated method used was failure-prone because of common artifacts in OCT images (e.g., detector saturation due to specular reflection).

Various extensions have been proposed to enhance the contrast in OCT images in general. For instance, compression OCT elastography (OCE) shows promise as a tool for interoperative assessment of cancer margins for *ex vivo* human breast samples.^12^ However, *in vivo* OCE imaging in the oral cavity with controllable tissue compression remains an intriguing prospect, which has not yet been reported. Polarization-sensitive OCT (PS-OCT) has demonstrated polarization contrast between uninvolved stroma and tumor^13^^,^^14^ and, by contrast, does not require tissue compression. Various studies using PS-OCT on healthy and pathologically altered animal^15^ and human^16^^–^^20^ oral tissue samples have revealed promising enhanced contrast provided by differences in the polarization properties of oral cavity tissue structures. An important recent advance is the refinement of reconstruction methods to enable extraction of local tissue birefringence (localized in depth), which has led to marked improvements in contrast and fidelity with tissue morphology, including in (healthy) oral cavity tissues.^21^

To further advance our understanding of contrast assessed by PS-OCT and its potential in oral cancer diagnosis and treatment, a detailed comparison with corresponding histology is needed. PS-OCT measurement directly after excision is not always feasible and investigation of fixed samples at a later time, if feasible, may be preferred. Work by Grieve et al.^22^ reported that tissue fixation may diminish the visibility of some features when measured with OCT. That study, however, investigated the fixation of retina samples and was performed with OCT intensity without insight into the polarization effects of the fixation process. No studies on the effects of tissue fixation on PS-OCT imaging have been presented to date. The closest research that we have found on the effects of fixation on tissue optical polarization properties was performed with optical polarimetry.^23^ Thin sections of porcine liver and heart were measured in transmission mode and an overall increase of birefringence upon fixation was reported. However, the authors cautioned that polarization contrast for other tissue types could not be extrapolated from their study and should be determined independently.

In this study, the effect of tissue fixation on local birefringence contrast determined with PS-OCT was assessed. *Ex vivo* oral cavity samples were measured immediately postexcision, then fixed in formalin solution and measured again 24 h after the commencement of fixation, following standard pathology. Some samples were measured 48 h after fixation to confirm the results. To the best of our knowledge, this is the first time such an investigation of PS-OCT imaging contrast has been performed. The results suggest that tissue fixation does not substantially alter local birefringence contrast and open up opportunities for broader studies based on a variety of pathological precancerous and cancerous oral tissue samples. Such studies should improve prospects for the *in vivo* differentiation of normal and abnormal tissue based on PS-OCT.

## 2 Materials and Methods

### 2.1 Specimen Preparation

The imaging was performed at the Oral Medicine Clinic, Sir Charles Gairdner Hospital (Perth, Western Australia). The study was approved by the Human Research Ethics Office of the University of Western Australia (UWA Ethics number RA/4/1/8562) and written informed consent was obtained from all participants. Over a 3-day period, we measured 12 freshly excised tissue samples from different locations in the oral cavity and covering a range of pathologies (see Table 1 for the clinical presentation of excised samples). Excised samples were transferred from the surgery to a nearby office and imaged with PS-OCT within 5 min of excision. Following the PS-OCT measurement, samples were immersed in a fixative solution (10% neutral-buffered formalin) and stored at room temperature. The measurement of all stored samples was repeated at 24 h from excision (±45  min). In routine pathology processing of *ex vivo* oral cavity tissue, samples of the size used in this study undergo 24-h fixation prior to further processing; therefore, we assumed 24 h was sufficient elapsed time for samples to be fully fixed. For reference, Hsiung et al.^24^ reported 18 h as the time necessary to ensure complete tissue fixation for hamster cheek pouch samples. Additionally, for further confirmation of fixation status, five samples were also measured at 48 h after excision.

**Table 1:** *Ex vivo* samples used for the study described by site in the oral cavity and clinical presentation.

#	Site	Clinical presentation
1	Right ventral tongue	Squamous papilloma
2	Right buccal mucosa	Leukoplakia
3	Right soft palate	Leukoplakia
4	Right buccal mucosa	Oral lichen planus
5	RHS lateral tongue	Hyperkeratotic area adjacent to ulcer
6	Left buccal mucosa	Oral lichen planus
7	Lower left lip	Actinic cheilitis
8	Left buccal mucosa	Oral lichen planus
9	Left lateral tongue	Leukoplakia
10	Left ventral tongue	Leukoplakia
11	Right soft palate	Leukoplakia with pigmentation
12	Right lower lip	Actinic cheilitis

### 2.2 Polarization-sensitive OCT

Imaging was performed with a custom PS-OCT scanner developed in-house and used previously for needle-based measurements of breast samples^13^ and adapted recently for bulk optics-based measurements.^24^ The technical specifications of the scanner are summarized as follows. The system operated with an A-line repetition rate of 50 kHz at the center wavelength of 1310 nm and a scanning range of 110 nm. We employed passively depth-encoded polarization multiplexing and polarization-diverse detection to measure the full Jones matrix containing the cumulative polarization information of backscattered light after round-trip propagation through the sample.^26^ To provide sufficient imaging range for depth-encoded multiplexing, a custom-made sampling clock frequency-doubling circuit was used. The resolution of the system was 13  μm and 20  μm in tissue in the axial (assuming a tissue refractive index n=1.4^27^) and lateral directions, respectively. Single volumetric data sets consisting of 1000×1000 A-scans covering an area of 5.1×5.1  mm with a depth range of 3.5 mm in air were captured within 20 s for each sample. Further technical details on the instrument (not relevant to the current study) can be found in Refs. 13 and 24. The XY scan area was sufficient to image the whole sample in the lateral dimensions.

**Fig. 1:**
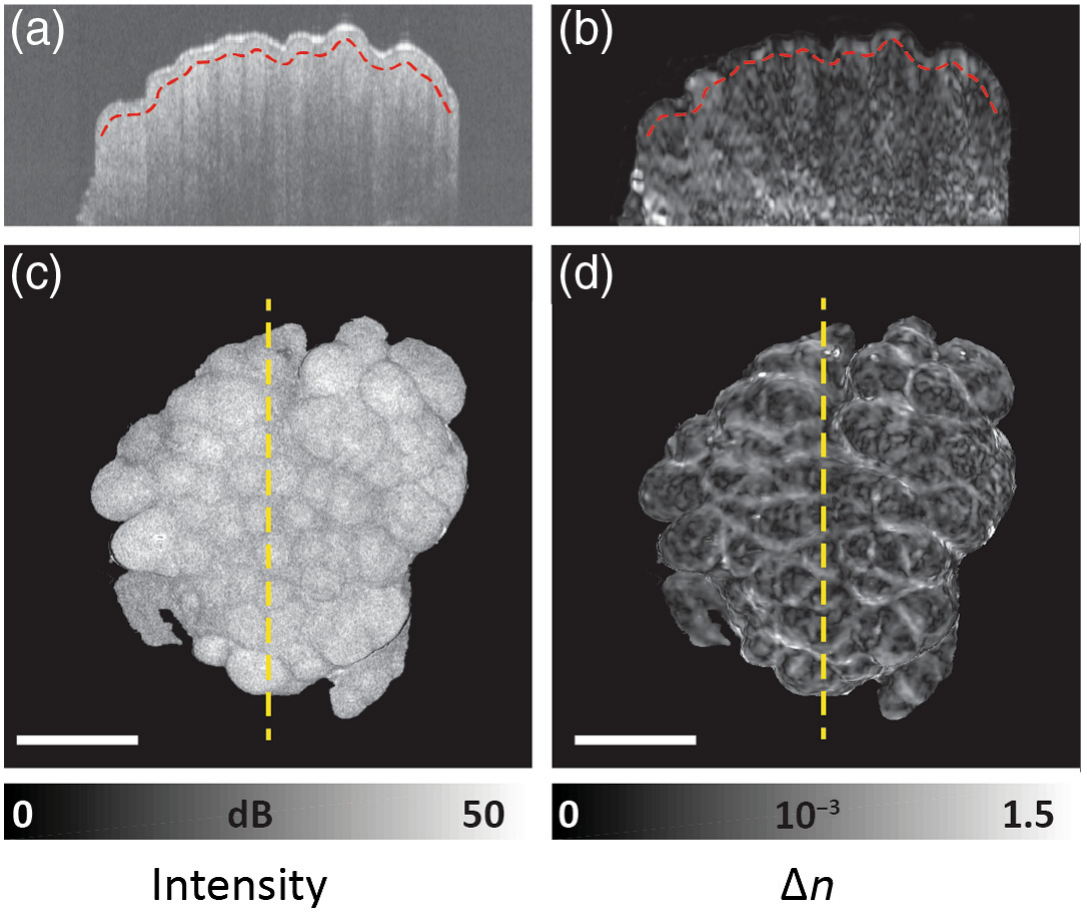
Cross-sectional (B-scan) PS-OCT images of the *ex vivo* right ventral tongue sample (sample #1 from Table 1): (a) conventional OCT intensity image, (b) depth-resolved birefringence (Δn) revealing increased contrast compared to OCT intensity image, (c) *en face* slices from the depth indicated by the red dotted curve in (a), and (d) depth-resolved birefringence from the depth indicated by the red dotted curve in (b). Yellow dotted lines correspond to the position of cross-sectional images. For images on (b)–(c), regions corresponding to low SNR, as measured in the conventional OCT images, were masked out. Images were scaled in depth assuming a refractive index of n=1.4. Scale bar: 1 mm.

Data processing to extract local birefringence is summarized as follows. Mueller–Jones matrices were constructed from the measured Jones matrices and spatially averaged before extraction of the local birefringence using a differential Mueller matrix algorithm.^13^ In contrast to the majority of previous studies, where average birefringence is computed from the cumulative retardance from the tissue surface to a given depth, here, we provide local birefringence at close to the same resolution as the OCT intensity images. Separately, in our recent *in vivo* study,^17^ we have shown that results presented by means of cumulative retardation provide lower contrast, especially at greater depths, compared with local birefringence. An averaging kernel of 26  μm axially and 40  μm laterally was used to improve the local birefringence contrast at the expense of a twofold reduction in spatial resolution. Both OCT intensity and local birefringence cross-sectional images are presented in grayscale in Figs. 1(a) and 1(b). Better insight regarding the features of measured samples is provided using an *en face* view [Figs. 1(c) and 1(d)]. The measured image data were first flattened by automatic subtraction of the extracted position of the tissue surface, so each *en face* slice corresponds to a given depth below the tissue surface (as marked by the red dotted curve on the cross-sections in Figs. 1(a) and 1(b). We note that, for some of the samples, the surface of the excised tissue does not correspond to the natural anatomical surface of the tissue before extraction. The flattening procedure was introduced to create *en face* slices and to enable automation of the quantitative analysis.

**Fig. 2:**
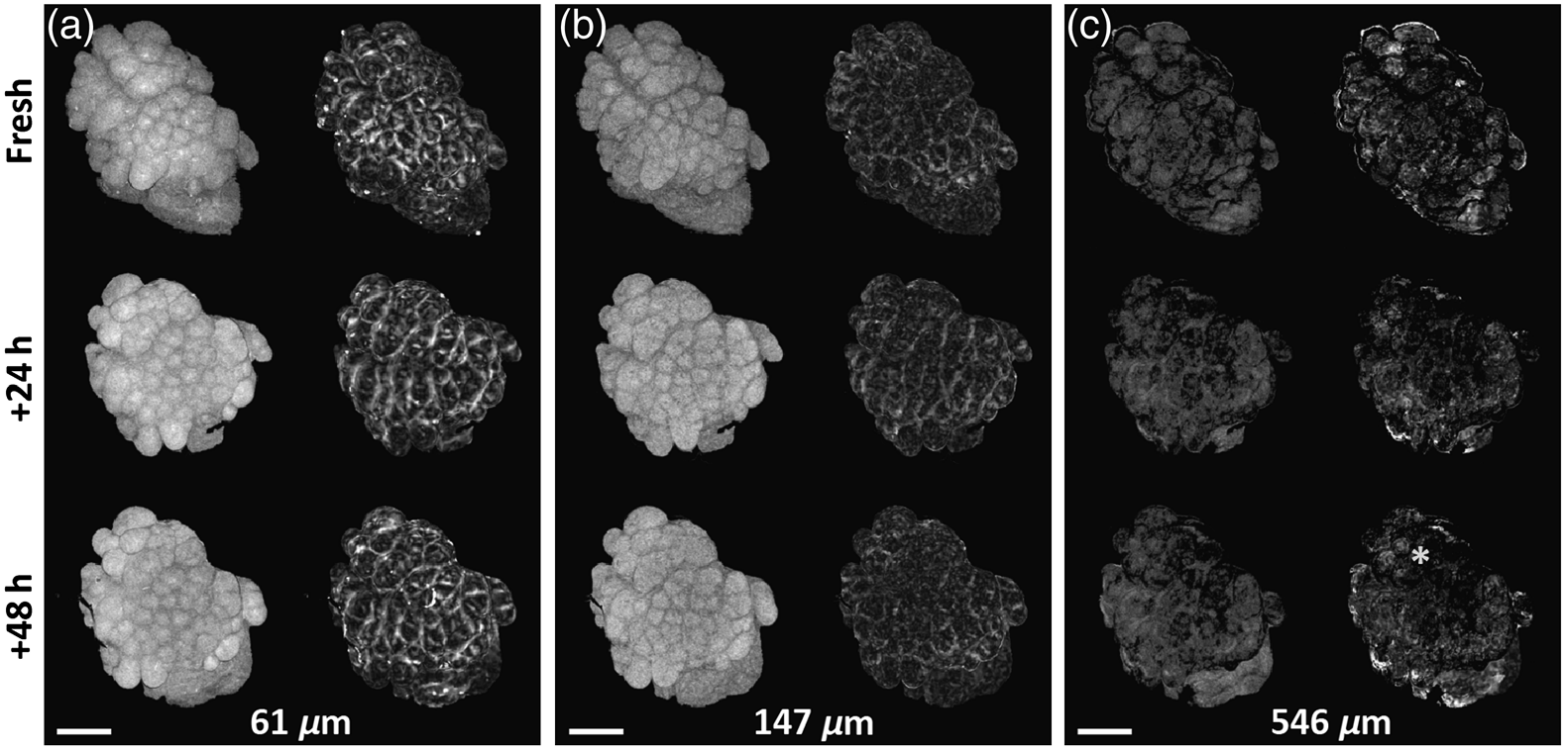
Paired—OCT intensity on the left and birefringence on the right—*en face* slices of an *ex vivo* right ventral tongue sample (sample #1 from Table 1) at various depths (a)–(c) for freshly excised tissue, and 24 h, and 48 h after fixation was initiated. Scale bar: 1 mm. The yellow asterisk indicates an area where birefringence contrast is locally reduced. The OCT intensity and birefringence scale bars are the same as in Fig. 1 and omitted here for clarity.

**Fig. 3:**
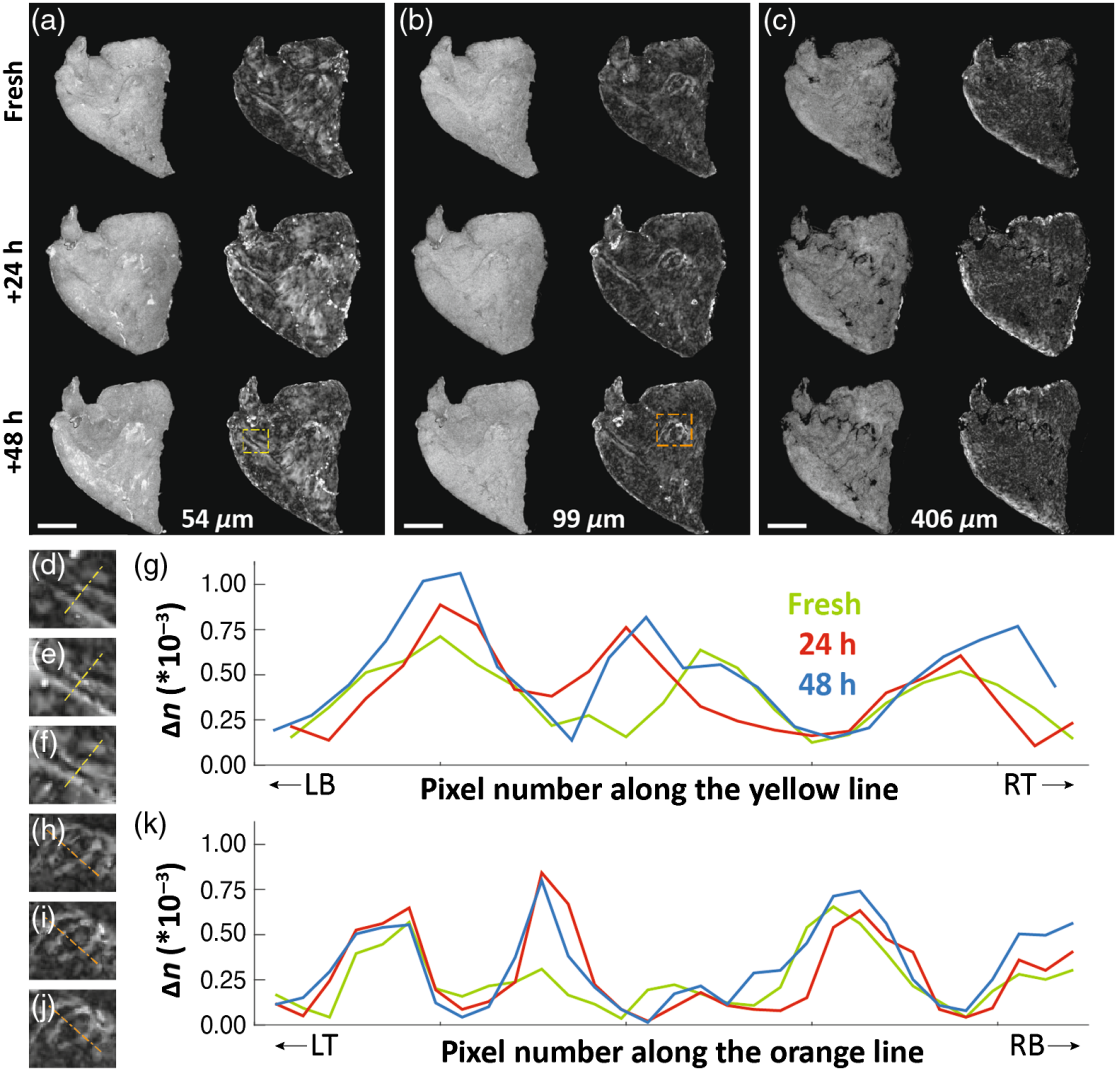
Paired—OCT intensity on the left and birefringence on the right—*en face* slices of the *ex vivo* right soft palate sample (sample #3 from Table 1) at various depths (a)–(c) for freshly excised tissue as well as 24 h and 48 h after fixation was initiated. The yellow and orange dashed-line boxes indicate the regions further visualized and analyzed in (d)–(k). Scale bar: 1 mm. Abbreviations used in (g) and (k): LB, left bottom and RT, right top related to the yellow dotted line on zoomed birefringence maps (d)–(f), LT, left top and RB, right bottom for similar orange dotted line (h)–(j). The OCT intensity and birefringence scale bars are the same as in Fig. 1 and omitted here for clarity.

Surface flattening was performed and *en face* slices were prepared for all measurements. The orientation angle (in the XY plane) of the same sample was found to differ between measurements for some samples. This was irrelevant for quantitative analysis of image contrast. However, for visual presentation and comparison purposes, the orientation angle difference was manually removed in postprocessing. The angle registration was performed by visual inspection based on sample topography features (see Figs. 1–3).

We automated the quantitative analysis of local birefringence contrast, thereby avoiding observer bias. We selected data for analysis using a binary mask (separate masks were created for each *en face* depth slice and each time point) by thresholding of OCT intensity data followed by two morphological operations: (1) closing of the image to fill the gaps in the mask and (2) erosion of the image to remove bright pixels not corresponding to the mask, applied to the mask to eliminate noise and irrelevant artifacts. The intensity-based masks were used on the corresponding *en face* slices of local birefringence. All procedures were optimized on one randomly selected data set, visually tested on three additional data sets, and then applied in unsupervised processing of all data sets. The contrast was calculated for each *en face* slice, starting from the surface to a depth of almost 800  μm, determined as the ratio of the standard deviation ($\sigma_{\Delta n}$) to mean value of birefringence ($\overset{¯}{\Delta n}$) (after application of the averaging kernel):

$$C_{\Delta n}=\frac{\sigma_{\Delta n}}{\overset{¯}{\Delta n}}.$$

For samples #9 and #11, the analysis depth was reduced to 600 and 450  μm, respectively, as no sample-related signal was present below these depths.

For each sample and time point, we performed a statistical comparison for every *en face* slice (N=112) between measurements of freshly excised samples and fixed samples measured 24 h (and 48 h where available) after the commencement of fixation. The statistical comparisons were first tested for normality of the distribution using the Shapiro–Wilk test.^28^ As all pairs were non-normally distributed, the Wilcoxon test^29^ with 5% significance level was used. Additionally, we analyzed the contrast differences at given depths in the complete sample population. For every depth slice, we calculated the contrast differences between the fresh and 24-h time points as well as between the fresh and 48-h time points. The mean difference and standard deviation for each slice were calculated and plotted versus depth.

## 3 Results

### 3.1 Local Birefringence Qualitative Contrast

Figure 2 shows *en face* OCT intensity and local birefringence sections extracted from different depths below the sample surface at the different time points of the fixation process. The sample was excised from the right ventral tongue and was preassessed as squamous papilloma. For this sample, we observe high variability of birefringent structures, which most likely represent highly aligned collagen fibers in the reticular layer, as described in our previous study.^21^ In general, in Fig. 2, we observe the contrast in both intensity and birefringence due to tissue alteration induced by the fixation procedure. For the deepest image sections [Fig. 2(c)], some examples of decreased contrast are observed (e.g., at the location marked by the yellow asterisk). This may be due to the effect of axial tissue shrinkage during fixation causing previously deeper layers to be shifted closer to the surface. The overall and rigorous assessment of the birefringence contrast change, however, is difficult based on visual inspection alone; therefore, we undertook the quantitative analysis described above and presented in the following sections.

The second example [Figs. 3(a)–3(c)] taken from the right soft palate reveals changes in the pattern of birefringent features. Magnified views [Figs. 3(d)–3(j)] depict visible improvement in the birefringence contrast during the fixation process. Two regions (yellow and orange boxes in Figs. 3(a) and 3(b) were selected to show the effect quantitatively. Zoomed maps of birefringence for freshly excised [Figs. 3(d) and 3(h)], 24 h [Figs. 3(e) and 3(i)], and 48 h [Fig. 3(f) and 3(j)] after fixation, as well as plots of birefringence extracted along selected lines [Figs. 3(g) and 3(k)], are presented. We chose to present data from the soft palate sample (Fig. 3) at different depths compared to the ventral tongue sample (Fig. 2) to help better appreciation of the variations in sample birefringence due to fixation, including with depth.

### 3.2 Local Birefringence Quantitative Contrast

**Fig. 4:**
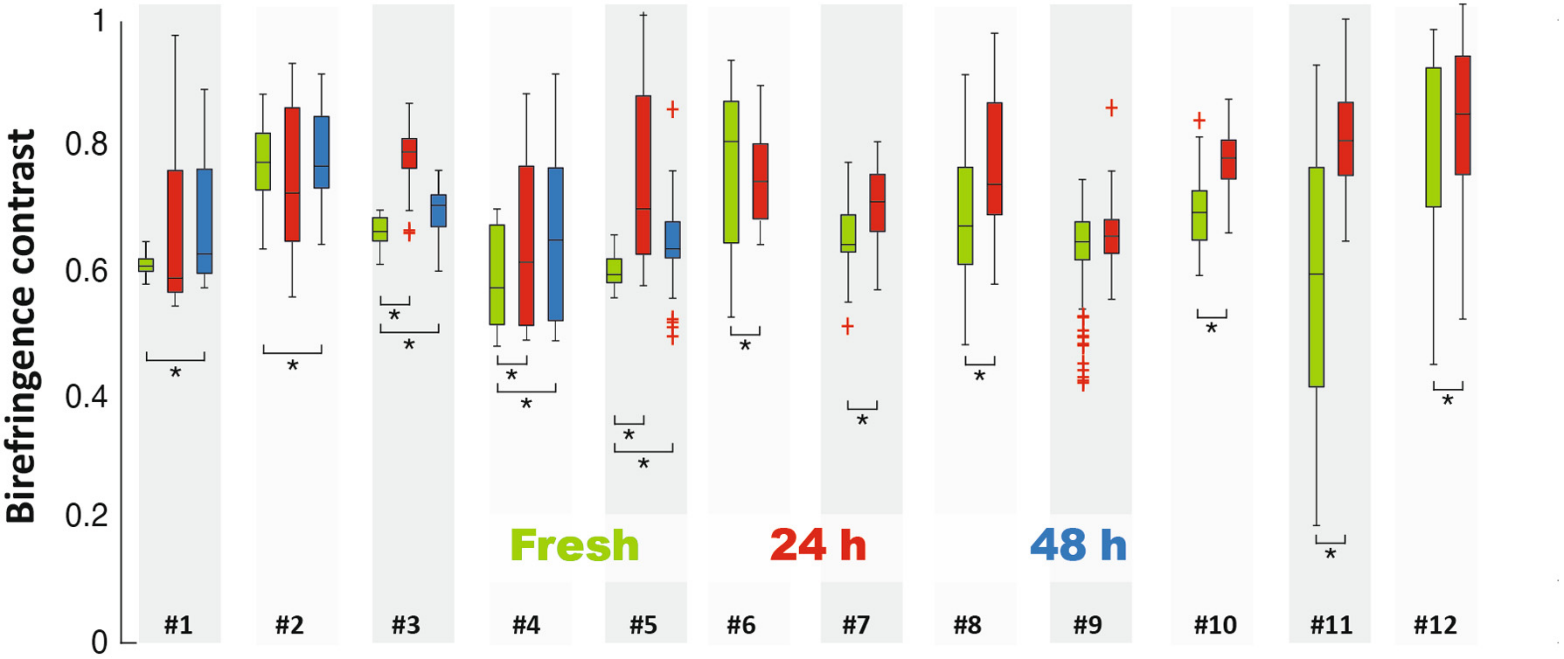
Quantitative comparison of contrast change during the fixation process for all measured samples (green color represents fresh; red color, 24 h after fixation was initiated; blue color, 48 h after fixation was initiated). Red crosses represent outliers (values more than 3 times the interquartile range away from the bottom or top of the box). Asterisks indicate statistically significant differences in 11 out of the 12 samples.

To provide more than the visual assessment of the effects of tissue fixation on polarization contrast, as described in Sec. 2, we applied automatic contrast analysis for both intensity and birefringence images. The results are presented as a boxplot (Fig. 4). For further statistical assessment, the paired t-test was applied to the results. Statistically significant differences were found for all cases except when fresh tissue was compared with 24 h after fixation for samples #1, #2, and #9.

**Fig. 5:**
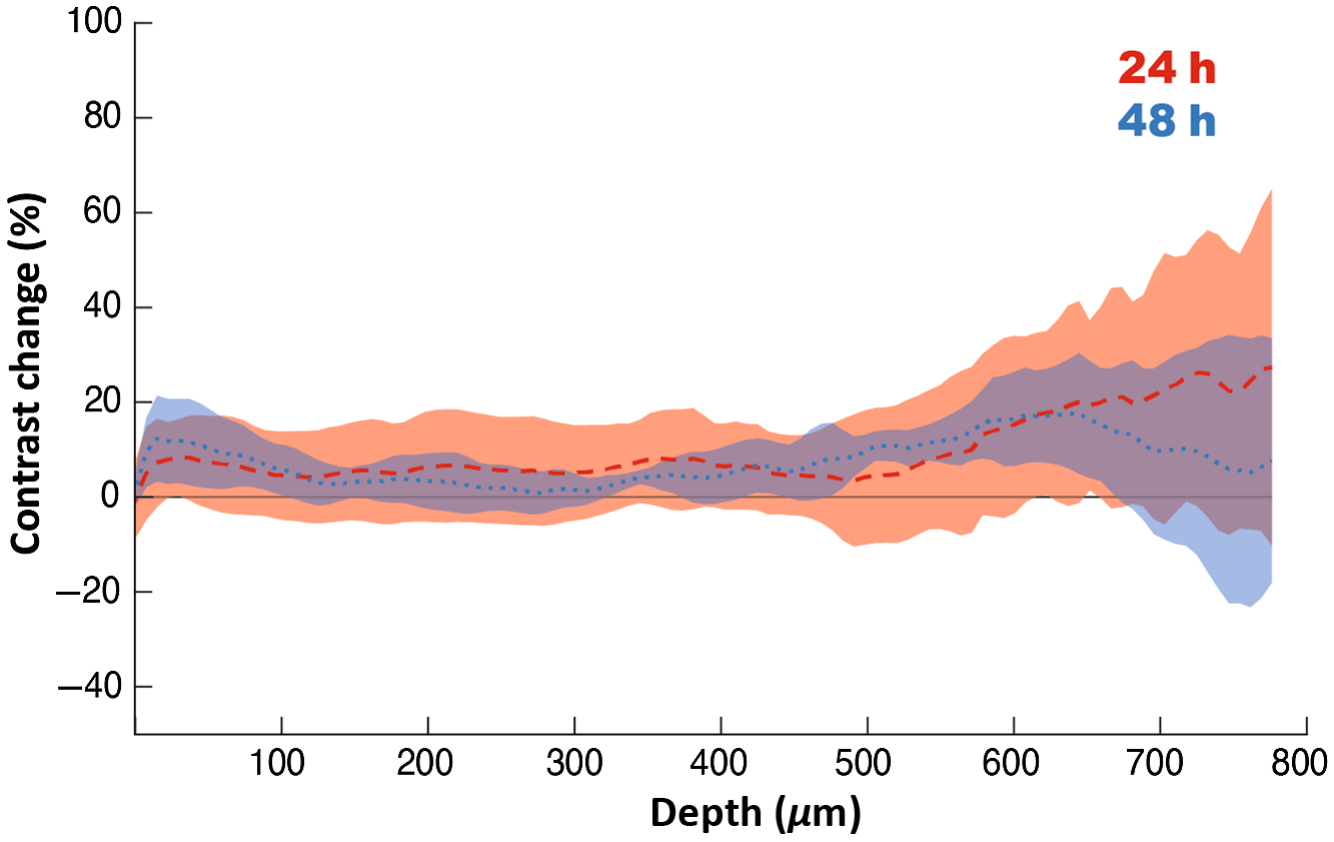
Change in fixed-sample contrast relative to fresh-sample contrast versus depth. Mean contrast change versus depth is presented with red-dashed curve for changes between fresh sample and 24 h after fixation, and blue-dotted curve for changes between fresh sample and 48 h after fixation. Corresponding ribbon plots represent ±1 standard deviation.

To provide more insight on how the contrast changes versus depth in the sample, we performed statistical (mean and standard deviation) slice-by-slice assessment of the differences in contrast between freshly measured samples and those measured 24 or 48 h after fixation. At each sample depth, the contrast change (24 h versus fresh and 48 versus fresh) was calculated. Depth by depth, we plot the mean contrast change over all samples with the ribbon representing standard deviation (Fig. 5). On average, we observe up to 10% increase in mean contrast to a depth of 500  μm followed by up to 20% increase for deeper layers. The variation, presented as a ribbon plot, is higher at deeper layers, where the SNR is usually lower than for preceding layers.

## 4 Discussion and Conclusions

In this study, the effect of fixation on local birefringence, assessed using PS-OCT, was investigated. Tissue samples from multiple locations in the oral cavity and covering a range of pathologies were imaged. Results presented in Fig. 4 show that tissue fixation does not diminish the contrast provided by the PS measurement; quite the contrary, the contrast is generally increased. Statistically significant increases in contrast were observed, compared to the contrast of fresh samples, in 11 of the 12 samples. The increase in mean contrast was calculated to be 11%. The greatest increases are observed in the deepest layers, which is most likely an artifact associated with lower ANR compared to superficial layers. The observed increase in birefringence with fixation represents a positive effect enhancing the utility of fixed oral cancer tissue samples.

A limitation of our work is that it does not explain the cause of the observed changes. In this study, however, our objective was to investigate whether local birefringence contrast is preserved after tissue fixation, and the extent to which fixation permits the assessment of fixed oral cavity tissues using PS-OCT. The observed effects may be the result of tissue shrinkage, which alters the patterns of birefringent structures in the measured samples. Such contrast enhancement may arise from the fact that local birefringence is calculated from the retardance 2kΔnδz across differential depth δz. In the case of axial shrinkage, differential depth δz reduces and leads to an increase in local birefringence, other things being equal. The unexpected result of differing contrast for some samples between measurements at 24 and 48 h after fixation raises questions that may be answered by variable sample shrinkage and related distortion, but which requires further investigation to be confirmed or otherwise.

Promisingly, from the perspective of the utilization of fixed oral cancer tissue samples, the enhancement of birefringent contrast $C_{\Delta n}$ removes the need to image samples immediately after excision. Furthermore, such relaxation of the need to perform imaging *in vivo* facilitates the future use of recently developed methods, including local optic axis imaging^25^ and ultrahigh-resolution, extended-focus PS-optical coherence microscopy (PS-OCM) that cannot yet be readily moved to a dental clinic.^30^ PS-OCM could offer more detailed insight into fine sample structure via imaging of the local optic axis, in sample regions with sufficient local birefringence.

It must be noted that even though the results of our work justify potentially bypassing measurements of freshly excited oral cavity samples, so they could be performed later beyond the clinical setting without loss of contrast, we are not proposing to displace *in vivo* measurements. In fact, *in vivo* measurements remain our main goal and we recently reported some interesting related *in vivo* results from healthy volunteers.^21^

A corollary to the above arguments is the conclusions drawn by the study of *ex vivo* samples will be more challenging to apply *in vivo*, in which images generally show lower contrast.

In the future, the study of numerous fixed samples might lead to processing methods, further enhancement of contrast and the emergence of clear means of differentiation between normal and abnormal tissue. Our results justify the use of fixed oral tissue in seeking a deeper understanding of birefringence contrast in oral tissue pathology; however, as mentioned previously by Wood et al.,^23^ we caution that similar investigations are advised if other tissue types are to be studied. The enhancement of contrast removes the need to image immediately postexcision and will facilitate exploration with other advanced tools, including mapping of the local optic axis with PS-OCT and ultrahigh-resolution PS-OCM.
